# Construction and validation of an anoikis-related prognostic model for lung adenocarcinoma based on bulk and single-cell transcriptomic data

**DOI:** 10.1371/journal.pone.0335788

**Published:** 2025-11-04

**Authors:** Yanfeng Xue, Yao Wang, Tianhao Huang, Yingjun Dong, Xin Tong

**Affiliations:** 1 Shanxi Province Cancer Hospital, Taiyuan, Shanxi Province, China; 2 Shanxi Hospital Affiliated to Cancer Hospital, Chinese Academy of Medical Sciences, Taiyuan, Shanxi Province, China; 3 Cancer Hospital Affiliated to Shanxi Medical University, Taiyuan, Shanxi Province, China; 4 Shanghai Institute of Measurement and Testing Technology Co., Ltd., Shanghai, China; 5 The First Affiliated Hospital of Soochow University, Suzhou, Jiangsu Province, China; The University of Texas, MD Anderson Cancer Center, UNITED STATES OF AMERICA

## Abstract

Lung adenocarcinoma (LUAD) is a highly aggressive lung cancer with poor prognosis due to lack of reliable biomarkers. Resistance to anoikis drives tumor progression and metastasis. This study aims to develop and validate an anoikis-related prognostic model for LUAD. We employed univariate Cox regression analysis, LASSO regression, and random forest algorithms to identify anoikis-related genes (ARG) from bulk transcriptomic datasets, and establish a 7-gene prognostic signature, validated in two LUAD cohorts from GEO database. We evaluated immune infiltration, molecular functions, and genomic alterations between risk groups and analyzed single-cell RNA sequencing data. IHC and mIF validated TIMP1 expression and its interaction with Treg cells. We developed a 7-gene prognostic model (LDHA, PLK1, TRAF2, ITGB4, SLCO1B3, TIMP1, ZEB2) using machine learning to predict survival in LUAD patients. The model accurately predicted 1-year survival rates (GSE31210: AUC = 0.805; GSE30219: AUC = 0.787), 2-year survival rates (GSE31210: AUC = 0.769; GSE30219: AUC = 0.681), and 3-year survival rates (GSE31210: AUC = 0.695; GSE30219: AUC = 0.735) and correlated with clinical features, immune infiltration, and tumor microenvironment (TME) remodeling. Single-cell sequencing data showed that LUAD patients exhibited an immunosuppressive TME phenotype, which was exacerbated by high TIMP1 expression in epithelial cells, promoting Treg cell activity. The 7-gene ARG prognostic model established in this study shows promising potential as a clinically applicable tool for decision-making.

## Introduction

Lung cancer exhibits the highest incidence and mortality rates among malignant tumors both in China and globally, with lung adenocarcinoma (LUAD) representing the most prevalent pathological subtype, accounting for approximately 40% of all lung cancer cases [1]. Current therapeutic strategies for LUAD encompass surgical resection, chemotherapy, targeted therapy, and immunomodulatory treatment [2,3]. Despite the evolution from conventional monotherapies to novel combination regimens, clinical outcomes including prognosis quality, metastatic risk, and recurrence rates have not demonstrated significant improvement, with the 5-year survival rate remaining at approximately 15% [4]. The prediction of clinical outcomes in LUAD remains a significant challenge due to its inherent prognostic heterogeneity and variable clinical courses [5–7]. This underscores the critical need to develop effective predictive models.

Anoikis, a programmed cell death mechanism, plays a pivotal role in determining the survival of cancer cells after their detachment from the extracellular matrix [8,9]. Substantial evidence indicates that anoikis serves as a crucial barrier against tumor metastasis by inhibiting the survival of circulating tumor cells and preventing their reattachment to new matrices [8]. For instance, studies have demonstrated that enhancing anoikis resistance in prostate cancer cells promoted their invasion and metastasis both in vitro and in vivo [9]. Similarly, CPT1A-mediated fatty acid oxidation facilitated colorectal cancer metastasis through anoikis suppression [10]. Consequently, the regulation of anoikis resistance represents a critical determinant in cancer progression [11,12]. Furthermore, anoikis-related genes exhibit prognostic potential in tumor progression. Mo et al. identified four prognostic anoikis genes (TLE1, GLI2, PLK1, and BAK1) and observed superior survival rates in patients with low anoikis scores (LAS) [13]. These findings suggest a significant association between elevated anoikis scores and poor prognosis in LUAD patients. While these insights represent important advances, the intricate relationship between anoikis resistance and LUAD prognosis warrants further investigation. Comprehensive studies remain essential to elucidate the nuanced mechanisms of anoikis resistance and their broader implications for optimizing LUAD management and therapeutic strategies.

In recent years, high-throughput transcriptomics has emerged as a powerful tool for deciphering disease complexity. Bulk RNA-seq provides an average gene expression profile of entire tissue samples, offering advantages such as cost-effectiveness and deep coverage, making it effective for identifying overall expression changes in disease states. However, its inherent “averaging” nature limits the ability to resolve cellular heterogeneity. In contrast, scRNA-seq resolves transcriptomes at single-cell resolution, enabling precise identification of cell subpopulations and characterization of their states. Nevertheless, scRNA-seq faces challenges such as limited sequencing depth, higher costs, and constraints in cell capture efficiency. Thus, integrating both approaches has become a compelling strategy to more accurately uncover cell-type-specific regulatory mechanisms during LUAD progression. This study elucidated the potential application of anoikis-related gene signatures obtained from TCGA and GEO databases for prognostic and diagnostic evaluation in LUAD patients and established a prognostic scoring model based on anoikis-related genes. Furthermore, we investigated differences in the tumor immune microenvironment among patients stratified by this prognostic model using scRNA-seq datasets. Importantly, our study identified TIMP1, a key modeling gene, as being highly expressed in epithelial cells of LUAD patients, which might exacerbate the immunosuppressive phenotype of the tumor microenvironment by promoting the proliferation, differentiation, and infiltration of Treg cells.

## Materials and methods

### Transcriptome data collection

Extensive RNA sequencing data, mutation data and clinicopathological characterization of TCGA-LUAD were downloaded from the UCSC Xena website (https://xena.ucsc.edu/). The RNA sequencing data included 520 LUAD samples and 59 normal samples, and the mutation data included 510 LUAD samples. These data were used as the model training set.

To assess the accuracy of the prognostic model in predicting the development of LUAD, we used 2 RNA sequencing data of LUAD patients from the GEO database as the model validation set, with the accession numbers GSE30219 and GSE31210.The GSE30219 dataset contained 67 LUAD samples, and the GSE31210 dataset contained 225 LUAD samples.

### scRNA-seq data collection

The scRNA-seq data of LUAD patients were obtained from GEO (https://www.ncbi.nlm.nih.gov/geo/, accession number GSE131907). It contained 11 cases of LUAD in situ tumors (Tumor group: GSM3827125-GSM3827135) and 11 cases of normal lung tissues (Normal group: GSM3827114-GSM3827124).

### The anoikis-related gene set collection

We extracted 434 anoikis-related genes (ARGs) with correlation scores > 0.4 from GeneCards (https://www.genecards.org/) and previous study results [14]. The specific gene set is shown in S1 Table.

### Differential expression gene analysis of transcriptome and enrichment analysis

Differential gene expression analysis was performed using the DESeq2 R package on TCGA-LUAD data comparing tumor versus normal tissues, with significance thresholds set at P < 0.05 and absolute log2 fold change (|log2FC|) > 1. The resulting differentially expressed genes (DEGs) were subsequently analyzed for functional enrichment using the clusterProfiler R package. Three gene sets – upregulated DEGs, downregulated DEGs, and all DEGs - were subjected to Gene Ontology (GO) analysis covering Biological Processes (BP), Cellular Components (CC), and Molecular Functions (MF), along with KEGG pathway and Hallmark gene set enrichment. Significantly enriched terms (P < 0.05) were selected, with the top 15 most significant pathways visualized (or all pathways if fewer than 15 met criteria).

### Construction and validation of prognostic models associated with ARGs

The intersection of DEGs from TCGA-LUAD data with ARGs yielded differentially expressed ARGs. To identify prognosis-associated candidate genes, we first performed univariate Cox regression analysis using the coxph function from the R package survival. Genes with a p value (wald) < 0.05 and a hazard ratio (HR) ≠ 1 were selected as preliminary candidates. Subsequently, LASSO regression (α = 1, 10-fold cross-validation) was performed with the glmnet and survival packages to filter the initial set down to 20 non-collinear candidate genes whose coefficients remained non-zero at the λ value giving the minimum cross-validation error. In parallel, random forest algorithm evaluated the importance of the initial 39 Cox-identified genes (The univariate Cox regression analysis results of these 39 genes are shown in S2 Table), retaining the top 10 most significant features, which carried out using the randomForestSRC package with the following parameters: ntree = 1000, nodesize = 10, and splitrule = ‘logrank’. The top 10 genes based on variable importance were selected. The intersection of genes selected by both LASSO regression and random forest methods identified seven core genes for prognostic model construction.

The calculation of the sample risk score was performed by summing the product of the risk coefficients of each gene and its expression value, calculated as:


$$\begin{array}{cc} h(t)= & \;ho(t)\times exp(ITGB4\times 0.0927+LDHA\times 0.306+PLK1\times 0.1992+SLCO1B3\times 0.121\\ & +\;TIMP1\times 0.1291+TRAF2\times 0.1165-ZEB2\times 0.1568) \end{array}$$

Two independent LUAD datasets (GSE30219 and GSE31210) from the GEO database were employed as external validation cohorts. ROC analysis was performed using the R package “pROC” (v1.18.5) to calculate AUC values and generate 1-, 2-, and 3-year ROC curves. The R “survival” package was utilized to assess prognostic differences between groups, with optimal risk stratification thresholds determined by the “surv_cutpoint” function. This approach partitioned samples into high-risk and low-risk groups, followed by Kaplan-Meier survival analysis to visualize and validate the model’s predictive performance.

### Construction and validation of nomograms

Prognostic evaluation of LUAD patients was performed through univariate and multivariate Cox regression analyses incorporating both the risk score and comprehensive clinicopathological characteristics, including age, gender (female/male), TNM staging (T1-4 for primary tumor extent, N0-3 for lymph node involvement, M0/1/MX for metastasis), smoking history (yes/no), and overall AJCC stage (I-IV). The analysis revealed that both the risk score and tumor stage emerged as independent prognostic factors in multivariate analysis (P < 0.01). Multivariate Cox analysis was conducted with the coxph function from the survival package. The optimal risk-score cutoff was determined using the surv_cutpoint function from the survminer package. Patients were classified into high- and low-risk groups accordingly. Kaplan–Meier curves were plotted and the log-rank *p*-value was computed using the survminer package.

To enhance clinical utility, we developed a prognostic nomogram using the rms package that integrated these significant predictors to estimate 1-, 3-, and 5-year survival probabilities.

### Immune infiltration analysis

The ESTIMATE algorithm was used to evaluate tumor purity and immune/stromal cell infiltration levels in high- and low-risk groups [15]. The Immune Score reflects immune cell infiltration, the Stromal Score indicates stromal cell infiltration, Tumor Purity represents the proportion of tumor cells, and the ESTIMATE Score combines immune and stromal evaluations. This method employs single-sample gene set enrichment analysis to generate these four scores.

Subsequently, we analyzed the 14 cancer-related functional status characteristics collected from the CancerSEA database [16]. The ssGSEA method with the R-package GSVA package was used to estimate the scores of the 14 cancer-related functional state features in each sample and to compare between high and low risk groups.

Finally, we analyzed 28 immune cell set features collected from the Pan-cancer database [17]. The ssGSEA method with the R-package GSVA package was used to estimate the relative abundance of the 28 immune cell set features in each sample and to compare between high and low risk groups. Spearman correlation was also used to analyze the correlation between TIMP1 and immune scores.

### Single-cell data quality control and cellular annotation

Single-cell RNA sequencing data were processed using the Seurat package, implementing stringent quality control measures: only genes detected in at least three cells were retained, and cells were excluded if they exhibited either <200 or >10,000 detected genes, < 1,000 total counts, or >20% mitochondrial/ribosomal gene content. Potential doublets were identified and removed using DoubletFinder. Batch effects were corrected via Harmony’s “RunHarmony” function, followed by standard Seurat workflows for normalization, clustering, and dimensionality reduction [18]. Specifically, the top 2000 highly variable genes selected by “FindVariableFeatures” were used for principal component analysis (20 retained PCs). Cell clustering was performed at resolution 0.6 (“FindClusters”), with UMAP visualization (“RunUMAP”). Cell populations were annotated based on CellMarker2.0 database and canonical marker genes.

### Screening of DEGs from single-cell data and functional enrichment analysis

We used the FindMarkers function in Seurat to identify DEGs for each cluster. Subsequently, functional enrichment analysis of these DEGs was performed using the R package “clusterProfiler” (v4.8.2), including Gene Ontology (GO) and Kyoto Encyclopedia of Genes and Genomes (KEGG) analysis [19].

### Trajectory analysis

Monocle (v2.28.0) was employed to construct the pseudotime trajectory based on the gene expression profiles of epithelial cells [20]. After dimensionality reduction and cell ordering, all epithelial cells were projected and ordered into trajectories with distinct branches, where cells within the same branch were considered to share an identical cell state. Branched Expression Analysis Modeling (BEAM) was further performed to identify genes exhibiting branch-dependent expression patterns. These branch-dependent genes contribute to exploring the mechanisms underlying cell fate determination.

### Analysis of intercellular communications

To investigate potential interactions between different cell types, we utilized the CellChat (v2.1.2) algorithm [21]. Merged Seurat objects containing epithelial cells and other cell types in the tumor microenvironment (TME) were used as input to the algorithm. After creating the CellChat objects, we built a reference database using the secretion signaling pathway. Specific receptor-ligand interactions and communication probabilities between different cell types were inferred using the computeCommunProb and computeCommunProbPathway functions, respectively.

### AUCell scoring

T cell subsets were analyzed for “Cytotoxicity” and “Progenitor exhaustion” functional gene sets using the AUCell package. The functional gene sets were derived from the TCellSI (T Cell State Identifier) method. T cell “Cytotoxicity” “Progenitor exhaustion” gene sets were analyzed. exhaustion T cell “Cytotoxicity” “Progenitor exhaustion” and functionally related gene sets are detailed in S3 Table.

### Patients and tissue samples

We included patients aged 18 years or older who underwent surgical resection for LUAD at The First Affiliated Hospital of Soochow University between January 1, 2023, and December 31, 2025. Paired tumor and paracancerous normal tissues were collected from each patient, all of whom had not received targeted therapy, radiotherapy, or chemotherapy prior to surgery. Tissue samples were fixed in 10% formalin and paraffin-embedded for standard histologic analysis.Data for this study were accessed on January 1, 2025, for research purposes. The authors did not have access to any personally identifiable information of the participants during or after data collection. Ethical approval for this study was obtained from the Ethics Committee of The First Affiliated Hospital of Soochow University Hospital (Ethics No. 2022−161). All procedures involving human participants were conducted in accordance with the ethical standards of the institutional and national research committees, as well as the Declaration of Helsinki and its later amendments or comparable ethical standards. Informed consent was obtained from all subjects.The patients in this manuscript have given written informed consent to the publication of their case details.

### Immunohistochemistry

Paraffin-embedded tumor or normal tissue specimens were sectioned into 4 μm-thick slices using a microtome, followed by dewaxing and antigen retrieval. Subsequently, endogenous peroxidase activity was blocked by incubating the sections with a peroxidase blocker (Beijing Zhongshan Jinqiao Biotechnology Co., Ltd., China) at room temperature for 10 minutes. The sections were then washed three times with PBS. After blocking with 10% goat serum, the sections were incubated overnight at 4°C with an anti-TIMP1 antibody (1:100 dilution, AF7007, Affinity Biosciences). Following three PBS washes, a secondary antibody was applied and incubated at 25°C for 30 minutes, followed by DAB staining for 10 minutes. Finally, the nuclei were counterstained with hematoxylin for 2 minutes, and the sections were examined under a microscope (Zeiss, Germany).

The immunostaining of TIMP1 in tumor and normal tissues was semi-quantitatively evaluated using the H-score system based on the percentage of positively stained cells and staining intensity. The staining intensity was categorized into four grades: negative (0), weak (1), moderate (2), and strong (3). The H-score for each case was calculated by multiplying the staining intensity by the corresponding percentage of positive cells, using the formula: H-score = (1 × % weakly stained cells) + (2 × % moderately stained cells) + (3 × % strongly stained cells). The resulting H-score ranged from 0 to 300.

### Multicolor immunofluorescence (mIF) staining

Tissue sections (4 μm thick) were dewaxed with xylene and rehydrated through a graded ethanol series (100%, 95%, and 70%). Antigen retrieval was then performed by microwave heating in citrate buffer for 15 minutes. Immunofluorescence staining was conducted according to standard protocols using TIMP1 (1:50, AF7007, Affinity Biosciences) and FOXP3 (1:100, ab20034, Abcam) antibodies. Sections were subsequently incubated with secondary antibodies at room temperature for 10 minutes. Between each staining cycle, heat-induced epitope retrieval was performed to remove all bound antibodies, including both primary and secondary antibodies. Multiplex immunofluorescence staining was carried out using the AlphaTSA Multiplex IF Kit (AXT37100041, AlphaX Biotech, China). Nuclei were counterstained with DAPI for 10 minutes before mounting with antifade medium. Multispectral images were acquired using a Zeiss AXIOSCAN 7 scanner (Zeiss, Germany). Quantitative analysis was performed using Fiji image analysis software based on the density and chromatic characteristics of immunostaining in the images.

### Statistical analysis

Statistical analyses were performed as follows: continuous variables were compared between two groups using the Mann-Whitney U test, while comparisons among three groups employed the Kruskal-Wallis test. Categorical variables were analyzed using the chi-square test for two-group comparisons. All statistical computations were conducted using R software (version 4.0.5), with a two-tailed P < 0.05 considered statistically significant.

## Results

### Screening of prognostic genes associated with anoikis‐related genes

**Fig 1:**
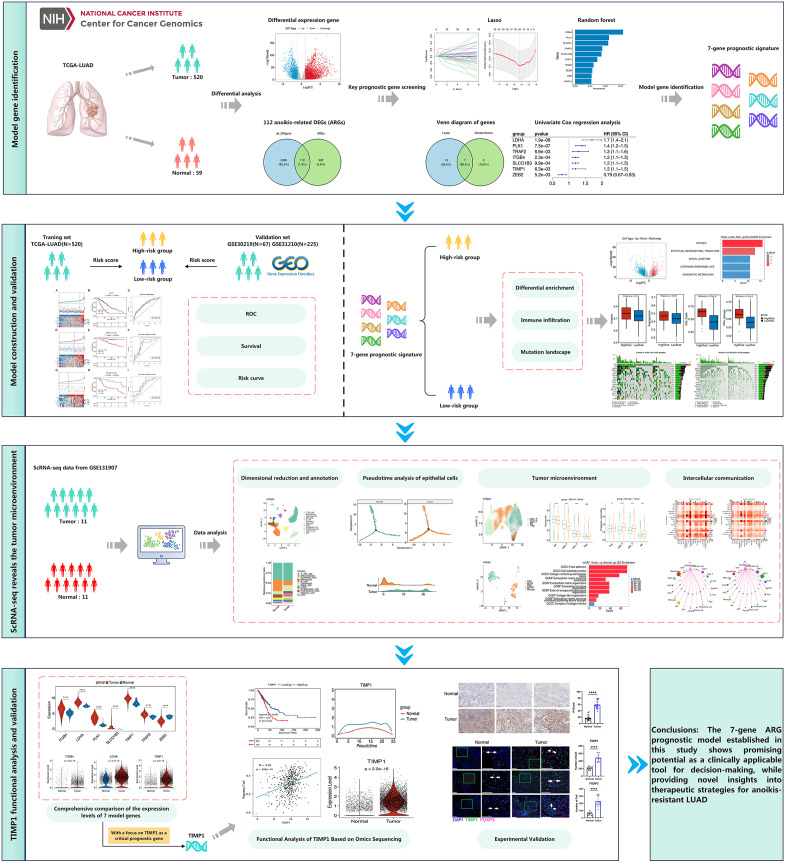
Workflow diagram for this study.

**Fig 2:**
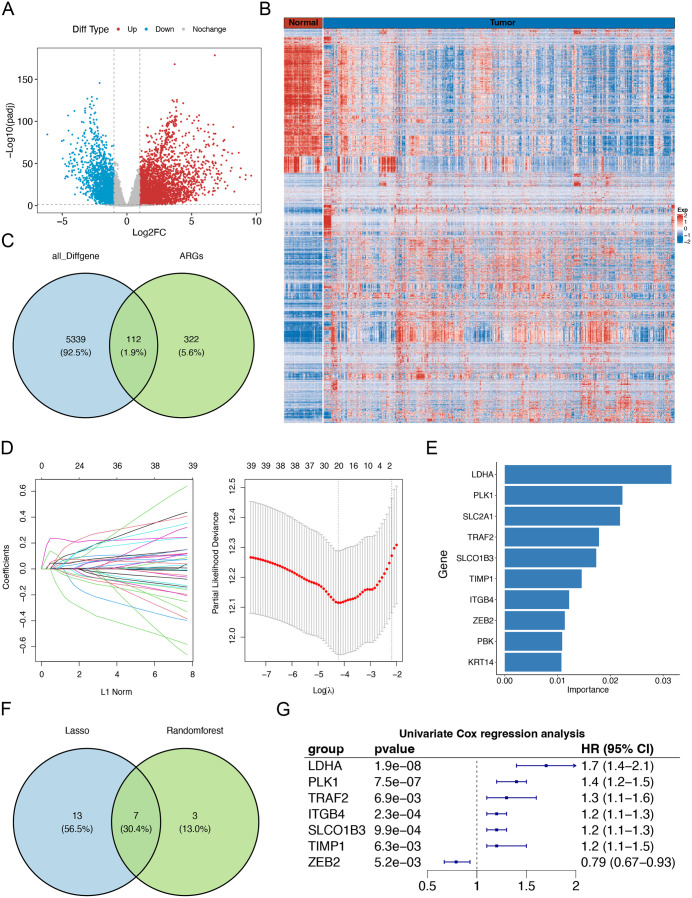
RNA sequencing analysis and screening of anoikis-related signature genes. (A) Volcano plot displaying differentially expressed genes (DEGs) between tumor and normal tissues based on TCGA-LUAD transcriptomic data. (B) Heatmap illustrating the expression patterns of all identified DEGs in TCGA-LUAD. (C) Venn diagram showing the intersection between all DEGs and anoikis-related genes (ARGs). (D) LASSO coefficient profiles (left) and cross-validation curve (right) for feature selection. (E) Random forest algorithm ranking the top 10 most significant prognostic anoikis-related genes identified by univariate Cox regression. (F) Venn diagram of genes selected by both LASSO and random forest approaches. (G) Forest plot presenting the univariate Cox regression results for the final signature genes.

The workflow of this study is shown in Fig 1. Based on the TCGA-LUAD transcriptome data, we analyzed and identified a total of 5,451 differentially expressed genes (DEGs), including 3,536 upregulated DEGs and 1,915 downregulated DEGs (Fig 2A and 2B). Subsequently, all DEGs were intersected with 434 anoikis-related genes (ARGs), resulting in a total of 112 anoikis-related DEGs (Fig 2C). univariate Cox regression analysis was conducted on these 112 genes identified 39 statistically significant anoikis-related DEGs (P < 0.05, hazard ratio ≠1) as potential prognostic biomarkers.

To identify core prognostic ARGs, we employed two distinct machine learning algorithms: LASSO regression and random forest analysis. LASSO regression analysis refined the initial 39 prognostic anoikis-related DEGs to 20 candidate genes (Fig 2D). Concurrently, random forest algorithm identified the top 10 most significant genes (Fig 2E). Further, through the intersection of prognosis-related ARGs filtered by LASSO regression and the random forest algorithm (Fig 2F), 7 core genes were identified for the construction of a prognostic model for LUAD. These genes were LDHA, PLK1, TRAF2, ITGB4, SLCO1B3, TIMP1, and ZEB2 (Fig 2G). Notably, LDHA, PLK1, TRAF2, ITGB4, and SLCO1B3 were identified as prognostic risk genes (P < 0.05, HR > 1), while ZEB2 emerged as a prognostic protective gene (P < 0.05, HR < 1).

**Fig 3:**
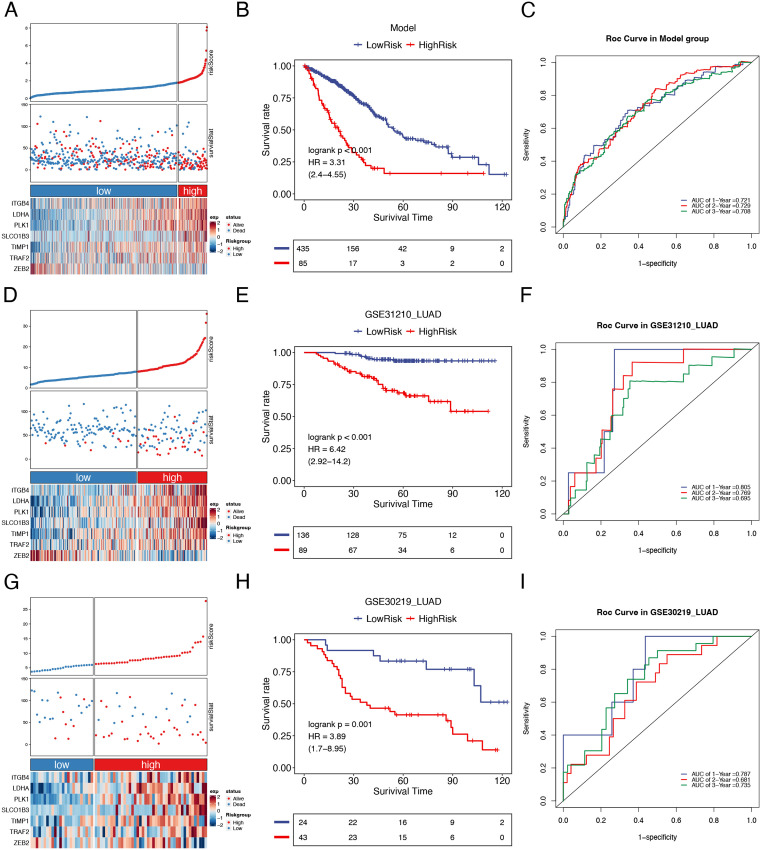
Construction and validation of a prognostic model for LUAD patients. (A-C) TCGA-LUAD training set (n = 520): (A) Risk score distribution, survival status, and signature gene expression patterns between high-risk (n = 85) and low-risk (n = 435) groups. (B) Kaplan-Meier survival curves comparing high- vs low-risk groups. (C) Time-dependent ROC curves assessing 1-, 2-, and 3-year survival prediction accuracy. (D-F) GSE31210 validation set: (D) Risk score analysis with corresponding survival status and gene expression profiles. (E) Kaplan-Meier survival curves comparing high- vs low-risk groups. (F) Time-dependent ROC curves assessing 1-, 2-, and 3-year survival prediction accuracy. (G-I) GSE30219 validation set: (G) Risk stratification with associated clinical outcomes and gene expression patterns. (H) Kaplan-Meier survival curves comparing high- vs low-risk groups. (I) Time-dependent ROC curves assessing 1-, 2-, and 3-year survival prediction accuracy.

**Fig 4:**
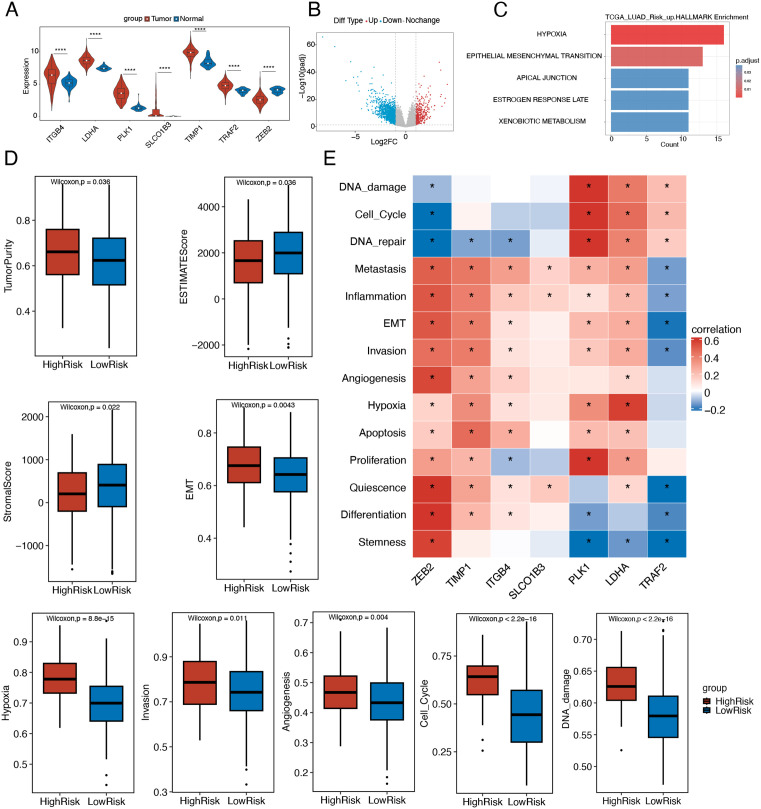
Analysis of differential enrichment and immune infiltration in high and low risk groups. (A) Box plots comparing expression levels of the 7 signature genes between tumor and normal tissues in TCGA-LUAD. (B) Volcano plot displaying DEGs between high- and low-risk groups. (C) Hallmark pathway enrichment analysis of upregulated DEGs in the high-risk group. (D) Box plots demonstrating differential cancer-related functional state scores between risk groups, as assessed using the CancerSEA database. (E) Heatmap depicting correlation patterns between the 7 prognostic signature genes and cancer-related functional state scores derived from the CancerSEA database.

**Fig 5:**
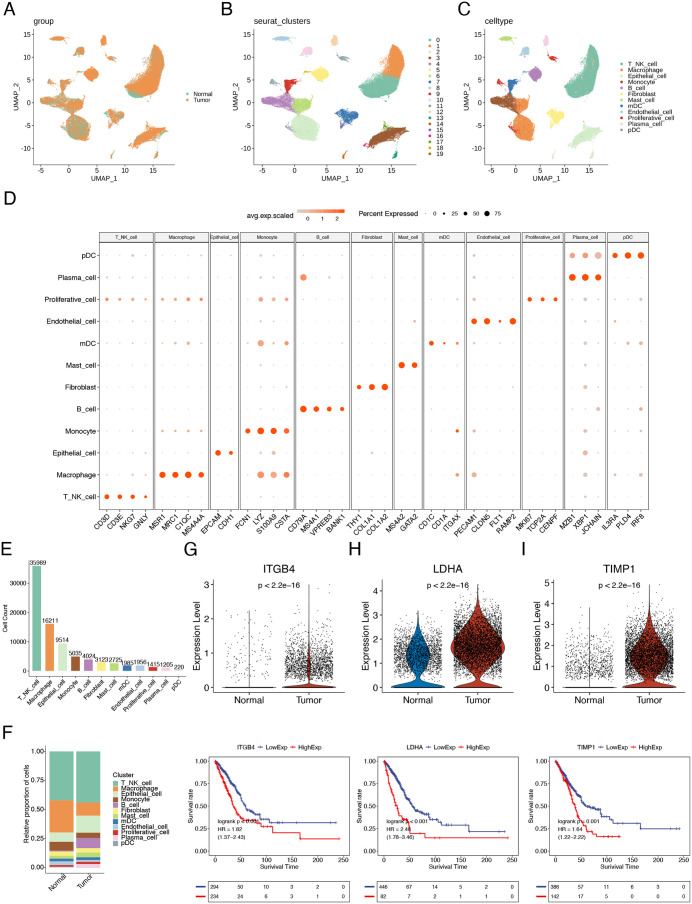
Single-cell landscape characterization in LUAD. Unsupervised clustering analysis of single cells was performed using UMAP visualization technology, with each dot representing an individual cell. (A) Coloring was conducted based on grouping. (B) Coloring was performed according to clusters. (C) Coloring was done based on cell types. (D) Characteristic marker genes of each cell type were displayed. (E) The quantity of different cell types was shown. (F) The proportion of cells in the two groups was presented. (G-I): The expression levels of key genes in epithelial cells from the tumor and normal groups, as well as the KM curves in the general transcriptome data of TCGA-LUAD, were illustrated for ITGB4 (G), LDHA (H), and TIMP1 (I).

**Fig 6:**
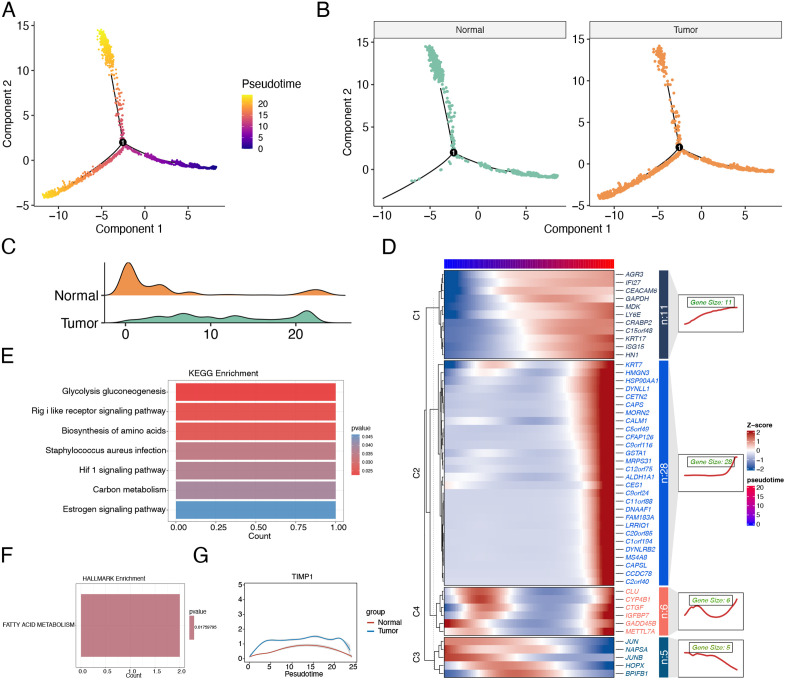
Temporal analysis of epithelial cells. (A-B) Epithelial cell trajectory and pseudotime analysis: (A) Cell trajectory colored by pseudotime order; (B) Cell trajectory colored by sample groups. (C) Ridge plot of cell differentiation states. (D) Heatmap showing dynamic gene expression changes along pseudotime. (E) KEGG pathway enrichment results for Cluster 1 genes. (F) HALLMARK pathway enrichment results for Cluster 2 genes. (G) TIMP1 expression trends along pseudotime in both groups.

### Construction and validation of a prognostic model for LUAD patients

To evaluate the efficacy and reliability of our prognostic risk prediction model, we first utilized the TCGA-LUAD cohort as the training set. Patients were stratified into high-risk (n = 85) and low-risk (n = 435) groups based on their risk scores. Significant differential expression of all 7 model genes was observed between these risk groups (Fig 3A). Kaplan-Meier survival curves and ROC curves were then generated by incorporating survival information for each sample. The Kaplan-Meier analysis demonstrated significantly shorter overall survival in high-risk patients compared to low-risk patients (P < 0.001, Fig 3B). ROC curve analysis of the training set yielded AUC values of 0.721, 0.729, and 0.708 for 1-year, 2-year, and 3-year overall survival predictions, respectively (Fig 3C). These results confirmed the model’s robust predictive performance.

Furthermore, our model was validated using 2 external datasets obtained from the GEO database (GSE31210 and GSE30219). Patients in each validation cohort were stratified into high-risk and low-risk groups based on the predetermined risk score cutoff. The results demonstrated significant differential expression of all 7 model genes between risk groups in both validation sets (Fig 3D and 3G). Kaplan-Meier analysis consistently revealed significantly worse overall survival in high-risk patients compared to low-risk patients in GSE31210 (P = 0.001, Fig 3E) and GSE30219 (P = 0.001, Fig 3H). Time-dependent ROC analysis yielded AUC values of 0.805, 0.769, and 0.695 for 1-, 2-, and 3-year overall survival predictions in GSE31210 (Fig 3F), and 0.787, 0.681, and 0.735 for corresponding predictions in GSE30219 (Fig 3I). These findings collectively demonstrate the robust prognostic performance of our model in effectively stratifying LUAD patients into distinct risk categories.

### Development of a survival rate prediction nomogram

To enhance the predictive model and facilitate clinical utility, we integrated multiple prognostic factors into a visual nomogram. Univariate Cox analysis identified risk score, gender, stage, T status, and N status as significant prognostic factors (P < 0.05, HR > 1; S1A Fig). Subsequent multivariate Cox analysis confirmed stage and risk score as independent predictors (P < 0.05, HR > 1; S1B Fig). Using the rms package, we constructed a nomogram to predict 1-, 3-, and 5-year survival probabilities for LUAD patients (S1C and S1D Fig). The model demonstrated strong predictive accuracy, with a concordance index (C-index) of 0.716 (S1C Fig). Calibration curves further confirmed superior agreement between predicted and observed outcomes compared to theoretical models (S1D Fig). These results collectively validate the nomogram’s reliability for clinical prognosis assessment.

### Analysis of differential enrichment and immune infiltration in high and low risk groups

We further analyzed the expression patterns of the 7 model genes in tumor tissues, revealing that TIMP1 exhibited the highest expression level among them (Fig 4A). Comparative analysis between high- and low-risk groups identified 1,825 DEGs, comprising 311 upregulated DEGs and 1,514 downregulated DEGs (Fig 4B). Hallmark gene set enrichment analysis demonstrated significant activation of hypoxia and epithelial-mesenchymal transition (EMT) pathways in the high-risk group (Fig 4C). Using the ESTIMATE algorithm, we evaluated tumor purity and stromal/immune cell infiltration across risk groups. The high-risk group showed significantly higher tumor purity scores, but lower stromal and ESTIMATE scores compared to the low-risk group (all P < 0.05), indicating greater stromal cell and immune cell infiltration in low-risk tumors. Furthermore, CancerSEA enrichment analysis revealed significantly elevated scores for cancer-associated functional states – including EMT, hypoxia, invasion, angiogenesis, cell cycle, and DNA damage – in the high-risk group (Fig 4D). These findings suggest that tumor cells in high-risk LUAD patients may possess enhanced invasive/metastatic capacities and greater immune evasion potential. Of particular interest, tumor epithelial cells appear actively involved in EMT, neovascularization, and invasion processes, indicating their likely pivotal role in driving the aggressive phenotype observed in high-risk patients.

We next performed correlation analysis between expression levels of the 7 model genes and cancer-associated functional state scores (Fig 4E). The results demonstrated that all 6 risk-associated genes (LDHA, PLK1, TRAF2, ITGB4, SLCO1B3, TIMP1) showed significant positive correlations with most functional state scores (P < 0.05, R > 0).

### Comparing mutation landscapes in high and low risk groups

Comprehensive analysis of TCGA-LUAD mutation data revealed that missense mutations and single nucleotide polymorphisms (SNPs) represented the predominant variant classification and type, respectively. The median mutation burden across samples was 162 variants, with the top 10 most frequently mutated genes being TTN, MUC16, CSMD3, RYR2, LRP1B, TP53, USH2A, ZFHX4, FLG, and KRAS (S2A Fig). When stratifying mutations by risk groups, waterfall plots displayed the top 20 mutated genes in each cohort. The high-risk group demonstrated a 95.12% mutation frequency (78/82 samples), compared to 90.42% (387/428 samples) in the low-risk group (S2B and S2C Fig). This pattern indicates significantly greater genomic instability in high-risk LUAD patients.

### Single-cell landscape characterization in LUAD

Single-cell data from 22 samples were acquired based on the scRNA-seq data of GSE131907, including 11 primary LUAD tumor tissues (tumor group) and 11 normal lung tissues (normal group) (Fig 5A). After rigorous quality control filtering, a total of 83,402 high-quality cells were retained for subsequent analysis. Dimensionality reduction and clustering partitioned these cells into 20 distinct clusters (Fig 5B), which were annotated as 12 cell types based on canonical marker gene expression: T/NK cells, macrophages, epithelial cells, monocytes, B cells, fibroblasts, mast cells, mDCs, endothelial cells, proliferative cells, plasma cells, and pDCs (Fig 5C and 5D). The three most abundant populations were T/NK cells, macrophages, and epithelial cells (Fig 5E). Comparative analysis revealed compositional shifts in the tumor group, characterized by reduced proportions of macrophages and monocytes alongside increased epithelial cell fractions (Fig 5F).

Our previous immune infiltration analysis revealed significant enrichment of tumor epithelial cell-associated pathways (EMT, invasion, angiogenesis) in the high-risk group (Fig 4D), highlighting the crucial involvement of epithelial cells in tumor progression. We therefore examined the expression of 7 model genes specifically in epithelial cells across tumor and normal tissues. Strikingly, only three genes - ITGB4, LDHA, and TIMP1 – showed significantly elevated expression in tumor epithelial cells compared to their normal counterparts (P < 0.01; Fig 5G–5I). Kaplan-Meier analysis confirmed worse prognosis for patients with high expression of these genes. Among these, TIMP1 emerged as particularly noteworthy, exhibiting the highest overall expression in tumor samples (Fig 4A) and strong positive correlations with multiple pro-tumorigenic functional states (Fig 4E). Previous studies have established TIMP1 as an oncogene that promoted malignant cancer phenotypes [22]. Furthermore, while TIMP1 is known to function as an anoikis suppressor [23], its specific mechanisms of regulating LUAD tumorigenesis and progression through anoikis modulation remain unclear. Based on these findings, our subsequent research will focus on investigating the regulatory mechanisms by which TIMP1 contributes to LUAD development and progression.

### Temporal analysis of epithelial cells

To further investigate the dynamic evolution of epithelial cells in tumor versus normal tissues, we performed trajectory analysis using the Monocle R package to determine cellular pseudotime distributions. The cell trajectory plots (Fig 6A and 6B) and ridge density plots (Fig 6C) revealed distinct temporal positioning between groups: epithelial cells from normal tissues predominantly occupied the early pseudotime phase of differentiation, while tumor-derived epithelial cells clustered primarily in intermediate and terminal differentiation stages.

Using the branching expression analysis model (BEAM), we identified 50 branch-dependent genes potentially critical in regulating cell differentiation (Fig 5D). These genes clustered into four distinct modules based on expression patterns: Cluster1/2 genes showed progressive upregulation along pseudotime, Cluster3 exhibited downregulation, while Cluster4 displayed dynamic “increase-decrease-increase” oscillations (Fig 6D). Functional enrichment revealed Cluster1 genes were predominantly associated with glycolysis, amino acid biosynthesis, carbon metabolism, and HIF1 signaling (Fig 6E), whereas Cluster2 genes enriched in fatty acid metabolism pathways (Fig 6F). These findings highlight metabolic reprogramming as a pivotal driver during normal-to-tumor epithelial cell differentiation, with likely cross-regulation between metabolic shifts and anoikis resistance. Notably, TIMP1 maintained consistently higher expression in tumor versus normal epithelial cells throughout pseudotime (Fig 6G), with its expression peaking during early-to-mid differentiation phases before declining. This temporal pattern suggests TIMP1, as a validated anoikis suppressor, may serve dual roles: (1) sustaining tumor epithelial cell proliferation by preventing ECM detachment during critical differentiation windows, and (2) facilitating metabolic adaptation through its peak expression coinciding with key differentiation transitions.

### Tumor microenvironment analysis

**Fig 7:**
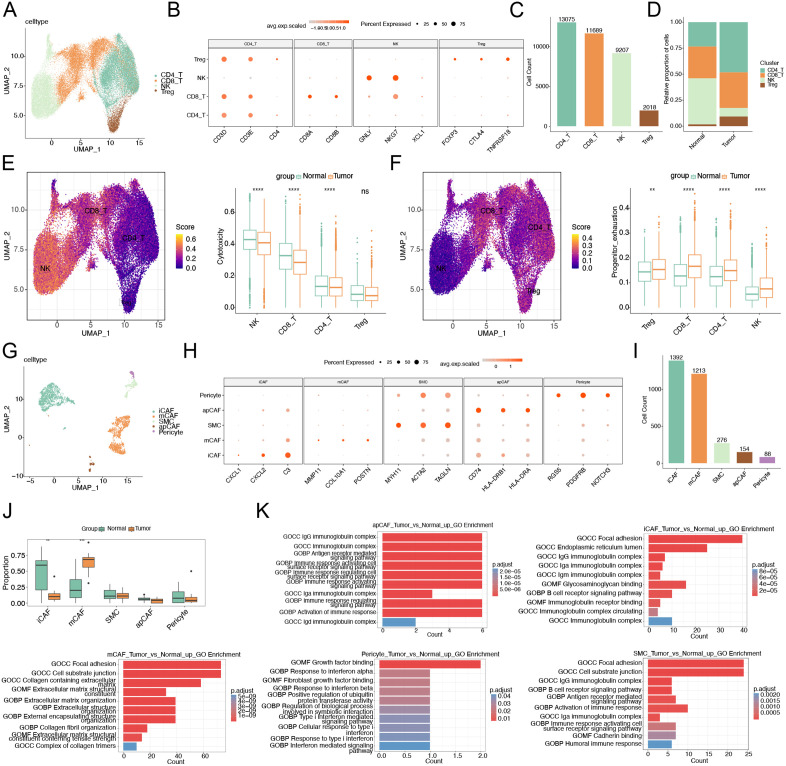
Tumor microenvironment analysis based on single-cell RNA sequencing results. (A) UMAP visualization of T/NK cell subpopulations. (B) Characteristic marker genes for each T/NK cell subset. (C) Bar plot showing cellular proportions of T/NK subpopulations. (D) Composition of T/NK cell subsets in tumor vs normal groups. (E) “Cytotoxicity” signature scores across T/NK subsets. (F) “Progenitor exhaustion” signature scores across T/NK subsets. (G) UMAP visualization of fibroblast subpopulations. (H) Characteristic marker genes for each fibroblast subset. (I) Bar plot showing cellular proportions of fibroblast subpopulations. (J) Composition of fibroblast subsets in tumor vs normal groups. (K) GO enrichment analysis of fibroblast subpopulations.

Given the critical role of tumor microenvironment (TME) in oncogenesis, we performed subclustering analysis of T/NK cells, identifying four distinct subsets: natural killer (NK) cells, CD4 + T cells, CD8 + T cells, and regulatory T cells (Tregs) (Fig 7A and 7B). Among them, CD4 + T cells represented the most abundant subset (Fig 7C). In addition, the percentage of NK cells in the tumor group decreased, while the percentage of treg cells increased (Fig 7D). This phenomenon suggests that the tumor group may exhibit a more immunosuppressive microenvironment. Functional scoring demonstrated marked impairment of cytotoxic potential in tumor-infiltrating lymphocytes, with significantly decreased “Cytotoxicity” scores in CD8 + T, CD4 + T, and NK cells compared to normal group (Fig 7E). Concurrently, all T/NK subsets exhibited elevated “Progenitor exhaustion” scores in tumor samples (Fig 7F), indicating widespread T-cell dysfunction. These findings collectively depict an immunosuppressed niche where T/NK cells exist in either inactive or exhausted states, exhibiting diminished tumoricidal capacity.

Fibroblast subclustering analysis identified five functionally distinct subsets: pericytes, antigen-presenting CAFs (apCAFs), smooth muscle cells (SMCs), myofibroblastic CAFs (mCAFs), and inflammatory CAFs (iCAFs) (Fig 7G and 7H). While iCAFs represented the most abundant population overall (Fig 7I), comparative analysis revealed significant compositional shifts in tumor tissues, characterized by increased mCAF proportions and decreased iCAF fractions (Fig 7J). Functional characterization uncovered unique pathway activation patterns for each subset: apCAFs showed enrichment in antigen presentation and immune regulation pathways; iCAFs in immunoglobulin complex and B-cell receptor signaling; mCAFs in extracellular matrix organization and collagen assembly; pericytes in interferon response and growth factor binding; and SMCs in immune response and cell adhesion pathways (Fig 7K).

These findings provide compelling evidence of tumor microenvironment remodeling, where reduced iCAF abundance correlates with diminished inflammatory responses – consistent with their known role in immune cell recruitment through cytokine secretion. Conversely, the expansion of mCAFs, which actively promote extracellular matrix remodeling, angiogenesis, and epithelial-mesenchymal transition, directly contributes to LUAD progression by fostering a tumor-permissive stromal niche. The observed shifts in CAF subset distribution (decreased iCAFs and increased mCAFs) reflect a coordinated transition toward a tumor-supportive microenvironment that facilitates matrix stiffening, promotes immunosuppression, and enhances angiogenic signaling – all hallmarks of aggressive tumor progression. These results provide specific insights into how distinct fibroblast subpopulations contribute to LUAD pathogenesis through their specialized functional programs.

### Analysis of intercellular communication

**Fig 8:**
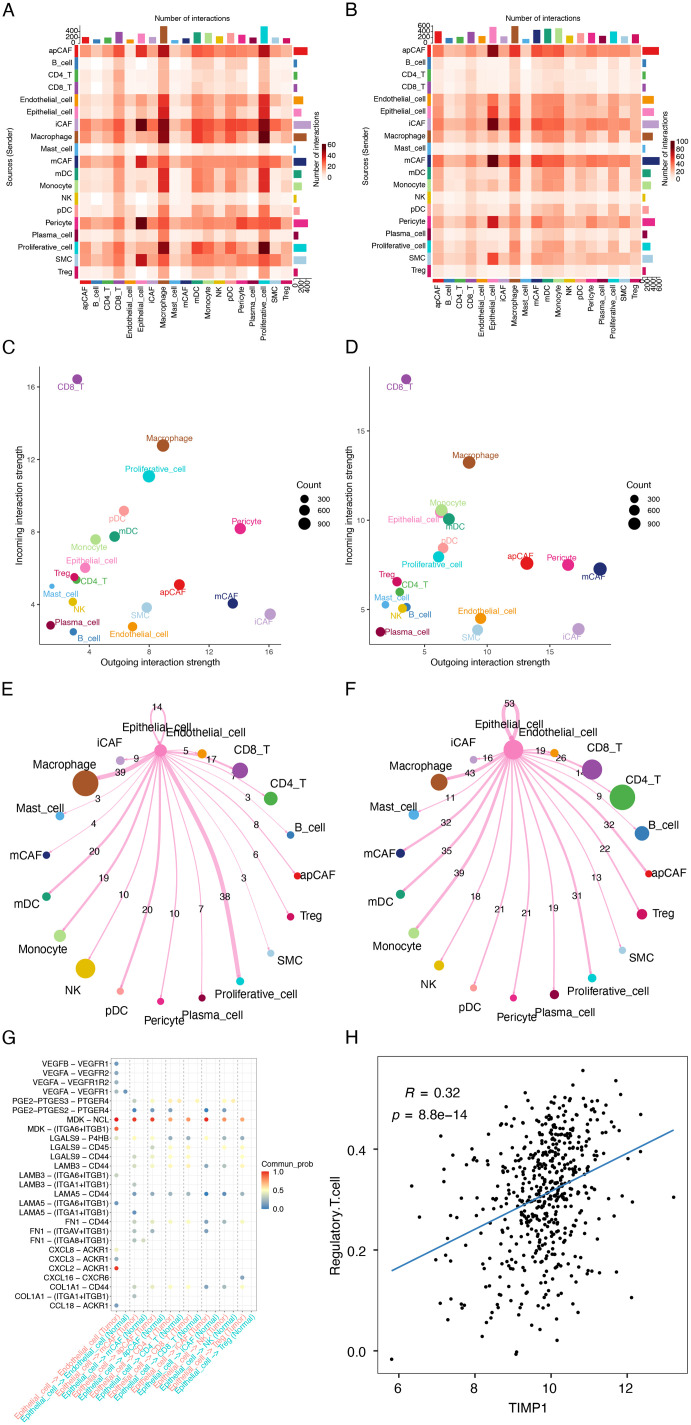
Cell-cell communication in the tumor microenvironment. (A-B) Total interaction numbers between specific cell subtypes innormal (A) and tumor groups (B). (C-D) Signal output/input strength across cell types in (C) normal (C) and tumor groups (D). (E-F) Chord diagrams depicting interaction networks between epithelial cells and other cell types in normal (E) and tumor groups (F). (G) Bubble plot comparing differential ligand-receptor interactions (epithelial cell as sender) between normal and tumor groups. (H) Scatter plot showing TIMP1 correlation with Treg cell abundance (Pan-cancer database).

To decipher intercellular communication within the TME, we constructed potential receptor-ligand interaction networks for both normal and tumor groups (Fig 8A and 8B). Comparative analysis revealed substantially altered communication patterns in tumor tissues, with epithelial cells exhibiting markedly enhanced signaling activity – demonstrating significantly increased signal-sending and signal-receiving capacities compared to their normal counterparts (Fig 8C and 8D).

Further analysis was conducted to examine the number of interactions and the ligand-receptor network relationships between epithelial cells (acting as signaling cells) and other cell types in both groups (Fig 8E–8G). The results revealed that in the tumor group, epithelial cells specifically interacted with endothelial cells through the VEGF signaling pathway by binding to the receptor VEGFR on endothelial cells, potentially promoting the formation of new blood vessels. Epithelial cells specifically interacted with Treg cells via the LAMININ pathway as well as through ligand-receptor pairs such as CXCL16-CXCR6, COL1A1-CD44, and FN1-CD44, which may facilitate the recruitment of Treg cells and contribute to an immunosuppressive microenvironment. Epithelial cells also specifically interacted with apCAFs, iCAFs, CD4 T cells, and CD8 T cells through the MDK, Prostaglandin, and GALECTIN pathways. The MDK pathway plays a role in promoting the proliferation and migration of malignant cells, while the Prostaglandin and GALECTIN pathways are involved in inhibiting the cytotoxic capacity of T cells and facilitating immune evasion. In addition, epithelial cells specifically interacted with mCAFs through the LAMININ, COLLAGEN, FN1, and MDK pathways, suggesting that in the tumor group, epithelial cells may promote cell proliferation and migration by attracting and recruiting mCAFs into the tumor microenvironment.

The observed expansion of Treg populations in tumor tissues (Fig 7D), coupled with the identified epithelial-Treg signaling networks, prompted systematic investigation of TIMP1’s immunomodulatory role. Analysis of Pan-cancer datasets revealed a significant positive correlation between TIMP1 expression and Treg infiltration levels in TCGA-LUAD samples (P < 0.05, R > 0.3; Fig 8H).

To delve into the mechanism by which TIMP1 regulates Tregs in epithelial cells of the tumor group, we categorized the epithelial cells in the tumor group into TIMP1-positive epithelial cell (TIMP1^+^Epi) and TIMP1-negative epithelial cell (TIMP1^-^Epi) subsets based on TIMP1 expression. Differential gene analysis of these two subsets revealed a significant upregulation of LGALS9 in the TIMP1^+^Epi subset (S3A Fig). LGALS9 is an immunosuppressive regulatory molecule that typically exerts immunosuppressive effects by binding to receptors on the surface of T cells. Studies have indicated that LGALS9 can enhance the immunosuppressive function of Tregs [24]. Further analysis of the number of interactions and ligand-receptor pairs between TIMP1^+^Epi cells and TIMP1^-^Epi cells as signaling cells interacting with Tregs revealed (S3B–S3D Fig): compared to TIMP1^-^Epi cells, TIMP1^+^Epi cells exhibited an increased number of ligand-receptor pairs interacting with Treg cells; moreover, TIMP1^+^Epi cells specifically regulated Treg subsets through the GALECTIN pathway (LGALS9-P4HB, LGALS9-CD45, LGALS9-CD44), the LAMININ pathway (LAMC1-CD44, LAMB3-CD44, LAMB5-CD44), as well as FN1-CD44 and COL1A1-CD44. Notably, the LAMININ pathway, along with FN1-CD44 and COL1A1-CD44, all play roles in cell adhesion, enabling the recruitment of Treg cells into the tumor microenvironment. Therefore, the high expression of TIMP1 in tumor epithelial cells promotes LGALS9 expression, which interacts with Treg cells through the GALECTIN pathway, thereby enhancing the immunosuppressive function of Tregs.

These results suggest a potential dual role for TIMP1 in LUAD progression: (1) as a direct promoter of epithelial cell survival through anoikis suppression, and (2) as an indirect facilitator of immune evasion by enhancing Treg recruitment and function within the tumor microenvironment. Therefore, we experimentally verified the infiltration relationship between TIMP1 and treg in tumor tissues.

### TIMP1 gene was highly expressed in LUAD tissues and regulated malignant cell- Treg interactions

**Fig 9:**
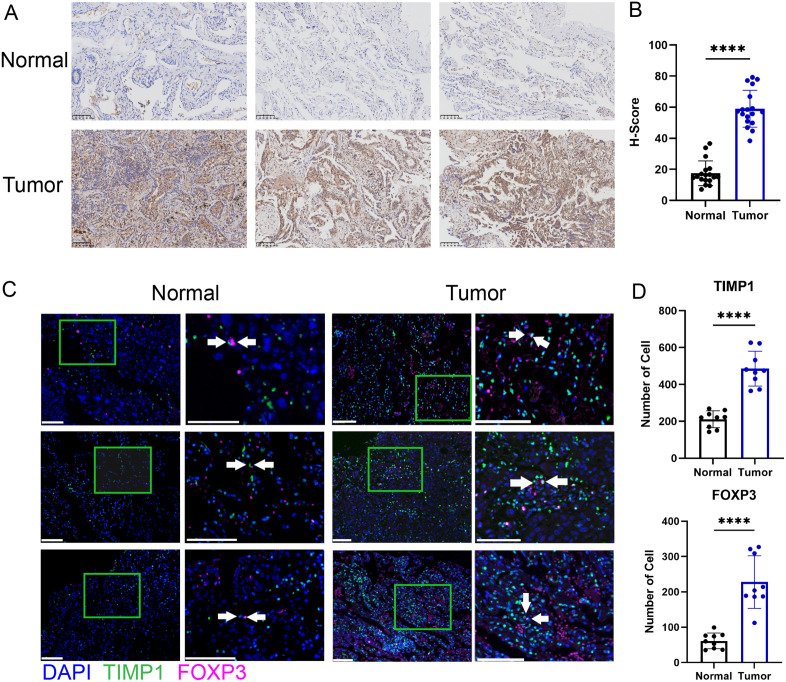
TIMP1 gene was highly expressed in LUAD tissues and regulated malignant cell- Treg interactions. (A) Representative immunohistochemical (IHC) staining of TIMP1 in normal (n = 8) and LUAD tissues (n = 8) (scale bar: 100 μm), Three fields of view were selected from each histopathological section. (B) Quantitative H-score analysis of TIMP1 staining intensity (normal vs LUAD). *p < 0.05; **p < 0.01; ****p < 0.0001. (C) Multiplex immunofluorescence (mIF) demonstrating spatial co-localization of FoxP3 + Tregs and TIMP1 + cells (scale bar: 100 μm). (D) Enumeration of TIMP1+ and FoxP3 + cells in normal (n = 3) and tumor tissues (n = 3), Three fields of view were selected from each histopathological section. *p < 0.05; **p < 0.01; ****p < 0.0001.

Subsequently, we validated TIMP1 expression patterns in LUAD tissues. Immunohistochemical (IHC) analysis demonstrated significantly higher TIMP1 expression in tumor regions compared to adjacent normal tissues (Fig 9A and 9B). To further elucidate the spatial relationship between malignant cells and Tregs and investigate whether TIMP1 directly regulates their interaction, we performed multiplex immunofluorescence (mIF) analysis of whole tumor sections. Among them, FoxP3^+^ cell populations were identified as Treg. The results revealed abundant Treg infiltration in LUAD tissues, with close spatial proximity between Tregs and TIMP1^+^/DAPI^+^ malignant cells (Fig 9C and 9D). These observations suggest potential functional interactions between malignant cells and Tregs that may be directly modulated by TIMP1, with significant implications for tumor microenvironment remodeling and immune response regulation.

## Discussion

Lung adenocarcinoma (LUAD) is the most common histological subtype of non-small cell lung cancer (NSCLC) [25]. Although significant progress has been achieved in LUAD treatment research, including advances in targeted therapy, immunotherapy, multidisciplinary approaches, and novel drug development, challenges persist in both treatment efficacy and prognosis [26]. Therefore, there is an urgent need to develop prognostic risk models and identify relevant biomarkers for LUAD.

Recent studies have highlighted the intricate interplay among non-apoptotic cell death pathways, which significantly influence the progression and therapeutic outcomes of various human cancers. These emerging cell death modalities, including autophagy-dependent cell death, ferroptosis, pyroptosis, and anoikis, offer promising therapeutic strategies for cancer treatment [27]. Metastasis occurs when localized tumor cells detach from the extracellular matrix (ECM) at their primary site [28]. The loss of contact with ECM or neighboring cells triggers anoikis, a specialized form of programmed cell death [29,30]. This process serves as a critical mechanism to prevent cell colonization and proliferation in new microenvironments [12]. Previous studies have primarily focused on the relationship between anoikis and tumor initiation, progression, proliferation, and invasion [31]. Notably, Mo Guiyan et al. investigated the association between anoikis-related genes and survival rates in LUAD patients [13]. However, no studies have yet established a risk prediction model for LUAD prognosis based on anoikis-related genes. In this study, we developed an anoikis-related gene signature to predict survival outcomes in LUAD patients using TCGA and GEO databases. Our analytical approach involved several key steps: First, univariate Cox regression analysis of 112 anoikis-related DEGs in LUAD patients identified 39 significant prognostic predictors. Subsequent LASSO regression and random forest analyses refined this to 7 core modeling genes (LDHA, PLK1, TRAF2, ITGB4, SLCO1B3, TIMP1, and ZEB2). Patients were stratified into high- and low-risk groups based on median risk scores. Notably, LDHA, PLK1, TRAF2, ITGB4, and SLCO1B3 were identified as risk genes (P < 0.05, HR > 1), while ZEB2 emerged as a protective factor (P < 0.05, HR < 1). Significant expression differences were observed for all 7 model genes between risk groups. Kaplan-Meier analysis confirmed poorer survival in high-risk patients, while ROC curves demonstrated the signature’s prognostic reliability. The nomogram results further suggested this risk score model may effectively predict LUAD survival status across different time points.

Differential gene expression analysis between high- and low-risk groups was subsequently performed, followed by enrichment analysis. The results revealed that genes in the high-risk group were predominantly enriched in cancer-promoting functional pathways, including epithelial-mesenchymal transition (EMT), invasion, and angiogenesis. Importantly, anoikis resistance was also implicated in malignant phenotypes such as EMT and metastasis. During metastasis, tumor epithelial cells normally undergo apoptotic cell death upon detachment from the primary site or during circulation. However, the acquisition of anoikis resistance – mediated through specific molecular mechanisms – enables these cells to develop migratory and invasive capabilities that facilitate metastatic dissemination [32,33]. These results suggest that the high-risk LUAD patients identified by our anoikis-based prognostic model likely exhibit enhanced metastatic potential due to anoikis resistance. Tumor epithelial cells appear to play a central role in this process, as evidenced by their active participation in EMT, neovascularization, and invasion – key features of the aggressive phenotype observed in high-risk patients. Mechanistically, anoikis occurs when epithelial and endothelial cells lose contact with the extracellular matrix (ECM) due to inadequate cell-matrix interactions in the tumor microenvironment [34]. Our observation that this process primarily affects epithelial cells informed our subsequent focus on this cellular population for further investigation. Furthermore, we performed comprehensive immune profiling comparing high- and low-risk groups. The analysis revealed significantly higher tumor purity scores in the high-risk group, coupled with lower stromal and ESTIMATE scores compared to the low-risk group. These findings suggest greater infiltration of stromal and immune cells in low-risk tumors. Importantly, these results indicate that tumor cells from high-risk LUAD patients may possess enhanced immune evasion potential, potentially contributing to their more aggressive clinical behavior.

To characterize the single-cell landscape of LUAD, we analyzed scRNA-seq data from GSE131907 and annotated 12 distinct cell types based on canonical marker gene expression. Epithelial cells emerged as one of the most abundant populations, with significantly increased proportions in tumor samples. Building upon our previous enrichment analysis showing high-risk groups were predominantly enriched in epithelial cell-related pathways (EMT, invasion, and angiogenesis), these findings underscore the pivotal role of epithelial cells in tumor progression. Consequently, we focused our investigation on the dynamic evolution of epithelial cells in tumor versus normal tissues. Our pseudotime analysis revealed distinct differentiation patterns: normal epithelial cells primarily occupied early pseudotime stages, while tumor epithelial cells clustered in intermediate and terminal differentiation phases. BEAM analysis further highlighted metabolic reprogramming as a key driver during the transition from normal to malignant epithelial phenotypes, suggesting potential crosstalk between metabolic shifts and anoikis resistance. This connection is biologically plausible, as existing evidence demonstrates intricate links between metabolic processes and anoikis regulation [35]. The initiation of anoikis itself depends on metabolic pathways, executed through both intrinsic (mitochondrial) and extrinsic (death receptor) caspase activation [36,37]. Conversely, anoikis-resistant cells exhibit profound metabolic alterations – for instance, PKA hyperactivation can stimulate autophagy and glutamine metabolism to mitigate ER stress and sustain energy production [38]. Notably, highly aggressive TNBC variants display metabolic dysregulation through glycolytic pathway activation; glycolytic inhibition has been shown to restore anoikis sensitivity in these cells [39]. Collectively, our findings suggest that tumor epithelial cell differentiation is profoundly influenced by anoikis-mediated metabolic reprogramming. This mechanistic understanding leads us to propose that targeting dysregulated metabolic pathways (e.g., glycolysis) may represent a novel therapeutic strategy to resensitize anoikis-resistant tumor cells. Such metabolic interventions could potentially overcome a critical barrier in cancer treatment by restoring normal apoptotic responses in malignant epithelial populations.

Our current investigation further examined the abundance patterns of the seven model genes in epithelial cell populations. Strikingly, only three genes—ITGB4, LDHA, and TIMP1—demonstrated significantly higher expression in tumor epithelial cells compared to their normal counterparts. Among these, TIMP1 emerged as particularly noteworthy, exhibiting the highest overall expression in tumor samples and showing strong positive correlations with multiple pro-tumorigenic functional states. This observation prompted focused investigation into TIMP1’s role in tumor progression. Pseudotime trajectory analysis of epithelial cells revealed that TIMP1 maintained consistently elevated expression throughout the entire differentiation continuum in tumor epithelial cells compared to normal cells. This persistent overexpression pattern suggests TIMP1 plays a more substantial role in epithelial cell differentiation within tumor tissues. Interestingly, TIMP1 expression displayed a biphasic pattern—initially increasing before subsequently declining—indicating its potential involvement during critical early, intermediate, and advanced phases of the malignant transformation process. TIMP1, a secreted protein best known for its inhibitory effects on matrix metalloproteinase 9 (MMP9), has been documented as overexpressed in multiple malignancies including lung, prostate, and thyroid cancers [22,40,41]—a finding consistent with our observations in LUAD. Mechanistically, TIMP1 functions as an anoikis resistance gene. In melanoma pathogenesis, it confered anoikis resistance through β1-integrin and CD63 interactions [42]. Our results suggest that during epithelial differentiation, TIMP1’s sustained high expression may protect tumor epithelial cells from ECM detachment, thereby facilitating their proliferation and differentiation. Furthermore, TIMP1 reduction has been shown to alter systemic and microenvironmental metabolism (e.g., lipogenesis and glycolysis), ultimately impairing tumor growth and survival [43]. These findings position TIMP1 as a dual-function regulator in malignant epithelial differentiation—both through direct anoikis resistance and by promoting metabolic adaptation. Nevertheless, in the seven-gene model, the remaining four genes—PLK1, TRAF2, SLCO1B3, and ZEB2—though not consistently expressed in tumor epithelial cells, also exhibit either oncogenic or tumor-suppressive functions. For example, targeting PLK1 has been shown to enhance the sensitivity of pancreatic cancer to immune checkpoint therapy [44]. TRAF2 promotes angiogenesis and cancer progression in renal cell carcinoma by facilitating the infiltration of M2-polarized tumor-associated macrophages [45]. SLCO1B3 acts as a tumor suppressor by inhibiting the development and progression of breast cancer [46], while ZEB2 promotes the growth and metastasis of liver cancer [47]. Collectively, the integration of these seven genes provides a more comprehensive predictive framework for assessing LUAD progression.

In recent years, cancer genomics has been undergoing a paradigm shift driven by artificial intelligence (AI). For instance, contrastive learning-based neural network frameworks—such as the Predictive Biomarker Modeling Framework (PBMF)—enable automated, systematic, and unbiased exploration of potential predictive biomarkers [48]. Patients identified with such biomarkers showed a 15% reduction in survival risk compared to those in the original trial cohort [48]. Moreover, the emergence of large-scale cancer foundation models, particularly transformer-based architectures pre-trained on vast multi-omics datasets (e.g., TCGA), has facilitated the learning of universal tumor biological representations. These models demonstrate strong transfer learning capabilities and exceptional performance in downstream tasks such as prognosis prediction and drug response [49,50]. The seven-gene prognostic signature identified in this study was developed within the context of this technological transformation. Consistent with previous reports, each of these genes has been implicated in regulating cancer progression. Furthermore, certain genes exhibit unique immunological functions—such as roles in immune regulation—that may complement other genes in the model, thereby enhancing its predictive accuracy for tumor progression.

Regulatory T cells (Tregs) play a pivotal role in LUAD progression by suppressing anti-tumor immunity and facilitating immune evasion [51]. However, enhancing the efficacy of tumor immunotherapy and improving its predictive value remain major clinical challenges. Notably, anoikis-related genes significantly influence Treg immunomodulatory functions, thereby affecting disease progression. For instance, pro-survival (anti-anoikis) genes were enriched in Tregs and dendritic cells, fostering an immunosuppressive TME [52]. Our study revealed an increased Treg proportion in tumor tissues, where epithelial cells specifically interact with Tregs to promote their infiltration into the TME. Consistent with prior research, the anti-anoikis gene TIMP1 exhibited a strong positive correlation with Treg abundance. These findings suggest a dual oncogenic role for TIMP1 in LUAD: (1) TIMP1 overexpression in epithelial cells inhibits anoikis, promoting their proliferation and differentiation; (2) TIMP1 enhances Treg expansion, differentiation, and recruitment, exacerbating the TME’s immunosuppressive phenotype and facilitating immune escape. Furthermore, we also found that TIMP1 expression was significantly correlated with Treg infiltration (R = 0.32, p < 0.001). However, the precise molecular mechanisms by which TIMP1 regulates Treg cells remain to be fully elucidated. TIMP1 has been reported to interact with the CD63-integrin complex, potentially leading to activation of the canonical TGF-β signaling pathway [53]. Given that TGF-β is a key cytokine driving the differentiation of naïve T cells into induced regulatory T cells (iTregs) [54], we hypothesize that TIMP1 may enhance local TGF-β bioactivity, thereby promoting Treg differentiation and function. Furthermore, recent studies have indicated that Notch and Wnt signaling pathways are also involved in modulating the stability and suppressive function of Treg cells [55,56]. As a multifunctional matricellular protein, TIMP1 may indirectly regulate these signaling cascades—possibly through interactions with cell surface receptors such as LRP1 [57]—to influence the transcriptional program of Tregs. Future studies employing both in vitro and in vivo models will be essential to validate these hypotheses and elucidate the precise mechanisms through which TIMP1 contributes to immune homeostasis. To validate this mechanism clinically, we employed multiplex immunofluorescence (mIF) on patient samples, revealing spatial proximity between TIMP1 + epithelial cells and Tregs. This spatial association suggests direct or paracrine interactions whereby TIMP1 may remodel the immune-resistant TME. In recent years, immune checkpoint inhibitors (ICIs) have revolutionized cancer treatment; however, their overall response rates remain suboptimal. A key reason is the abundance of immunosuppressive cells, such as regulatory Tregs, within the tumor microenvironment. These cells facilitate immune escape and therapy resistance by suppressing effector T cell function. Therefore, identifying patient populations with high Treg infiltration—who are likely to exhibit resistance to ICIs—is critical for advancing precision immunotherapy. Our study suggests that the TIMP1-associated risk model or Treg enrichment signature developed here may serve as a potential tool for screening individuals who are less likely to benefit from ICIs. More importantly, patients identified as high-risk based on Treg abundance may represent ideal candidates for novel Treg-targeted therapies, such as anti-CTLA-4 antibodies, anti-CCR4 antibodies, CD25-directed agents, and other emerging modalities.

This study has several limitations that should be addressed in future research. First, as our findings were derived from retrospective analysis of public datasets, prospective studies are needed to validate the prognostic accuracy of our model. Second, the molecular mechanisms underlying TIMP1-mediated anoikis resistance and Treg infiltration require further elucidation through detailed mechanistic investigations. Third, the potential involvement of TIMP1 in specific metabolic pathways that induce anoikis resistance remains to be determined. Future studies incorporating experimental validation and multi-omics approaches will be essential to address these knowledge gaps and advance the clinical translation of these findings. Additionally, our study was limited to a single disease focus (LUAD only), and potential batch effects may have arisen during dataset integration, while prospective validation was also lacking. These issues should be addressed in future research.

In this study, we developed a prognostic model for LUAD patients by applying machine learning algorithms to anoikis-related genes. Our findings further demonstrated that the tumor microenvironment in LUAD patients undergone remodeling toward an immunosuppressive phenotype. Notably, the modeling gene TIMP1, which was highly expressed in epithelial cells of LUAD patients, appeared to exacerbate this immunosuppressive TME by promoting the proliferation, differentiation, and infiltration of Treg cells. Collectively, we have established an anoikis-related prognostic signature and identified TIMP1 as a potential anoikis-associated biomarker, providing valuable insights for predicting clinical outcomes in LUAD patients.

## Supporting information

S1 Fig Development of a survival rate prediction nomogram. (A) Univariate Cox regression analysis identifying potential prognostic factors. (B) Multivariate Cox regression analysis determining independent prognostic factors. (C) Nomogram integrating independent prognostic factors for clinical prediction. (D) Calibration curve assessing the nomogram’s predictive accuracy.
(PDF)

S2 Fig Comparing mutation landscapes in high and low risk groups. (A) Comprehensive mutational profile of LUAD patients from TCGA-LUAD mutation dataset. (B) Mutational landscape in high-risk patients. (C) Mutational landscape in low-risk patients.
(PDF)

S3 Fig Exploration of the mechanism by which TIMP1 + Epi regulates Treg cells. (A) Expression levels of each gene in immune cells, TIMP1^+^Epi subpopulation and TIMP1^-^Epi subpopulation. (B-C) Interaction network diagram illustrating the relationships between immune cells and signaling cells, with TIMP1^+^Epi cells or TIMP1^-^Epi cells serving as the signaling cells. (D) The quantity of interactions and ligand-receptor pairs between TIMP1^+^Epi cells and TIMP1^-^Epi cells as signaling cells and Treg cells.
(PDF)

S1 Table The list of 434 anoikis-related genes (ARGs). (XLSX)

S2 Table The univariate Cox regression analysis identified statistically significant anoikis-related DEGs (P < 0.05, hazard ratio≠1). (XLSX)

S3 Table The gene set related to the “Cytotoxicity” and “Progenitor exhaustion” function of T cells. (XLSX)
