# Correction: Construction and validation of an anoikis-related prognostic model for lung adenocarcinoma based on bulk and single-cell transcriptomic data

**DOI:** 10.1371/journal.pone.0340504

**Published:** 2026-01-05

**Authors:** Yanfeng Xue, Yao Wang, Tianhao Huang, Yingjun Dong, Xin Tong

The first two authors, Yanfeng Xue and Yao Wang, should be noted as contributing equally to this work.

## References

[1] Xue Y, Wang Y, Huang T, Dong Y, Tong X. Construction and validation of an anoikis-related prognostic model for lung adenocarcinoma based on bulk and single-cell transcriptomic data. PLoS One. 2025;20(11):e0335788. doi: 10.1371/journal.pone.0335788 41187171 PMC12585065

