# Supplementary material for: Construction and validation of an anoikis-related prognostic model for lung adenocarcinoma based on bulk and single-cell transcriptomic data
Source: PLoS One. 2025 Nov 4;20(11):e0335788. doi: 10.1371/journal.pone.0335788 (PMC12585065; doi:10.1371/journal.pone.0335788)
Supplement: S1 Fig — (A) Univariate Cox regression analysis identifying potential prognostic factors. (B) Multivariate Cox regression analysis determining independent prognostic factors. (C) Nomogram integrating independent prognostic factors for clinical prediction. (D) Calibration curve assessing the nomogram’s predictive accuracy. (PDF) [file pone.0335788.s001.pdf]

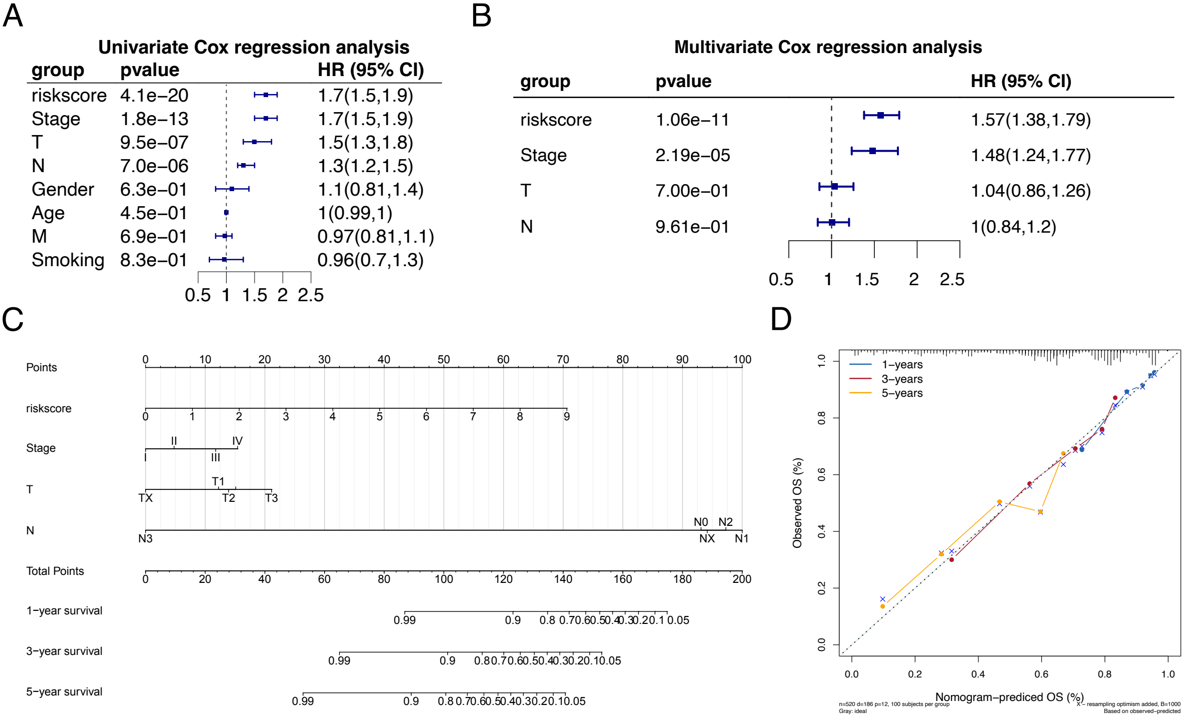
A
Univariate Cox regression analysis
group pvalue HR (95% CI)
riskscore 4.1e−20 1.7(1.5,1.9)
Stage 1.8e−13 1.7(1.5,1.9)
T 9.5e−07 1.5(1.3,1.8)
N 7.0e−06 1.3(1.2,1.5)
Gender 6.3e−01 1.1(0.81,1.4)
Age 4.5e−01 1(0.99,1)
M 6.9e−01 0.97(0.81,1.1)
Smoking 8.3e−01 0.96(0.7,1.3)
0.5 1 1.5 2 2.5
B
Multivariate Cox regression analysis
group pvalue HR (95% CI)
riskscore 1.06e−11 1.57(1.38,1.79)
Stage 2.19e−05 1.48(1.24,1.77)
T 7.00e−01 1.04(0.86,1.26)
N 9.61e−01 1(0.84,1.2)
0.5 1 1.5 2 2.5
C
Points
0 10 20 30 40 50 60 70 80 90 100
riskscore
0 1 2 3 4 5 6 7 8 9
Stage
I II III IV
T
TX T1 T2 T3
N
N3 N0 NX N2 N1
Total Points
0 20 40 60 80 100 120 140 160 180 200
1−year survival
0.99 0.9 0.8 0.7 0.6 0.5 0.4 0.3 0.2 0.1 0.05
3−year survival
0.99 0.9 0.8 0.7 0.6 0.5 0.4 0.3 0.2 0.1 0.05
5−year survival
0.99 0.9 0.8 0.7 0.6 0.5 0.4 0.3 0.2 0.1 0.05
D
1−years
3−years
5−years
Observed OS (%)
0.0 0.2 0.4 0.6 0.8 1.0
Nomogram−prediced OS (%)
n=520 d=186 p=12, 100 subjects per group
Gray: ideal
X − resampling optimism added, B=1000
Based on observed−predicted
