# Supplementary figures and images for: Construction and validation of an anoikis-related prognostic model for lung adenocarcinoma based on bulk and single-cell transcriptomic data
Source: PLoS One. 2025 Nov 4;20(11):e0335788. doi: 10.1371/journal.pone.0335788 (PMC12585065; doi:10.1371/journal.pone.0335788)

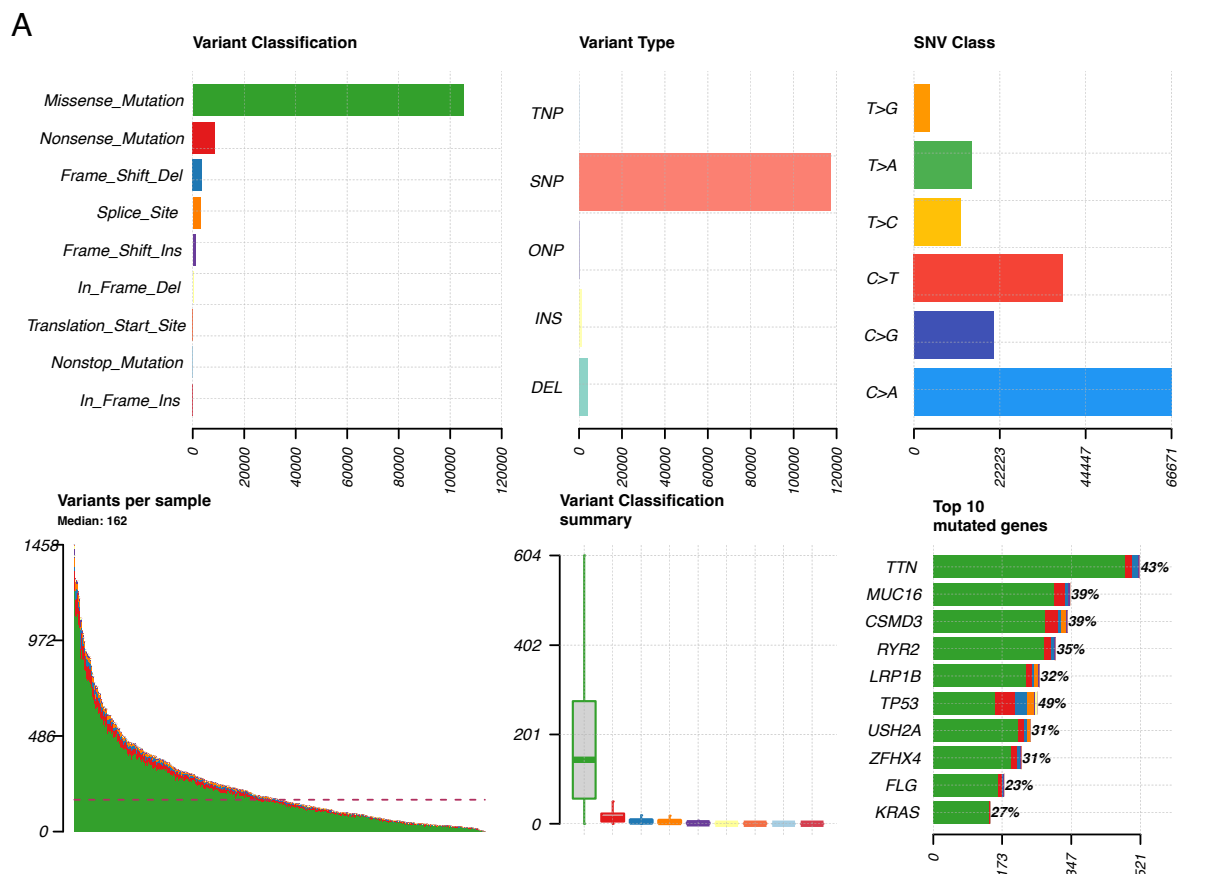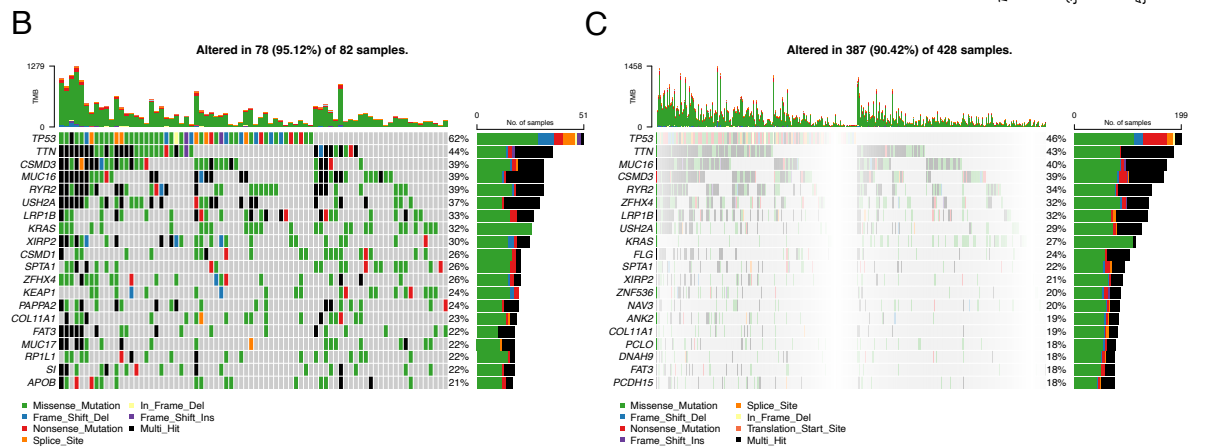

Supplement: S2 Fig — (A) Comprehensive mutational profile of LUAD patients from TCGA-LUAD mutation dataset. (B) Mutational landscape in high-risk patients. (C) Mutational landscape in low-risk patients. (PDF) [file pone.0335788.s002.pdf]

A

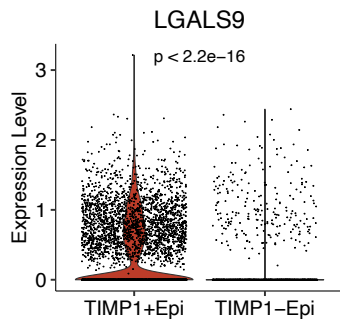

B

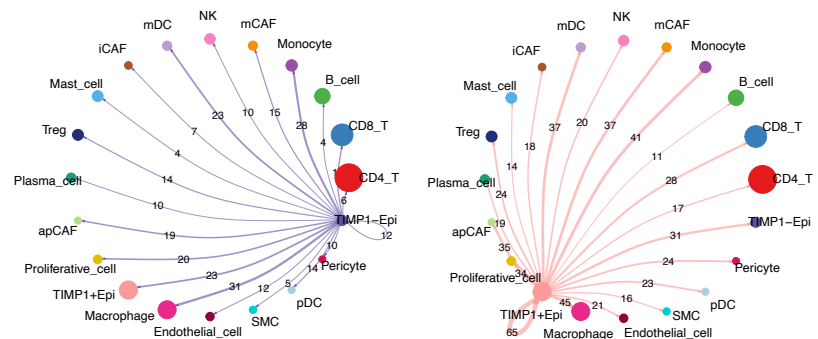

C

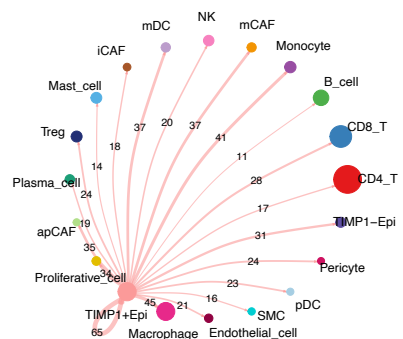

D

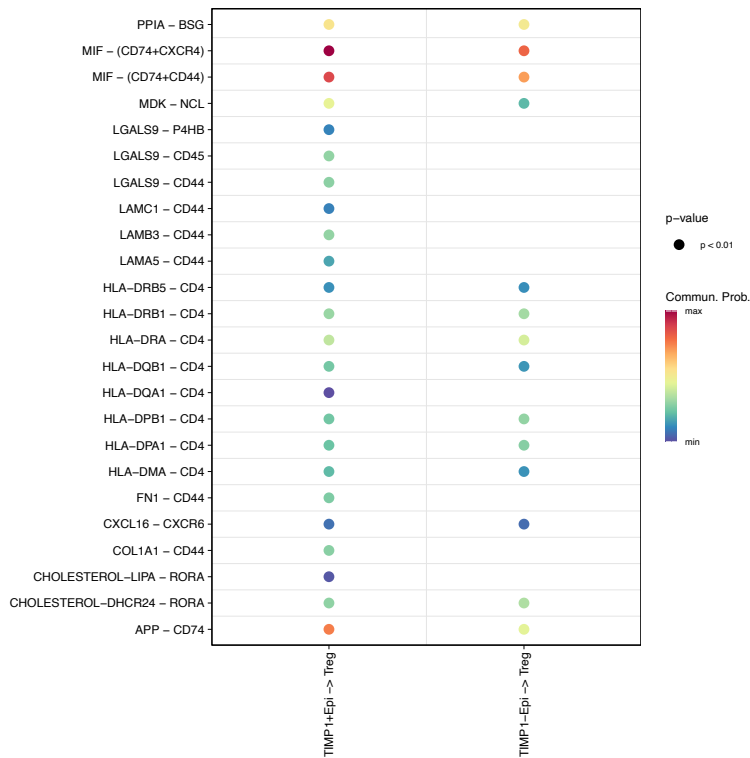

Supplement: S3 Fig — (A) Expression levels of each gene in immune cells, TIMP1+Epi subpopulation and TIMP1-Epi subpopulation. (B-C) Interaction network diagram illustrating the relationships between immune cells and signaling cells, with TIMP1+Epi cells or TIMP1-Epi cells serving as the signaling cells. (D) The quantity of interactions and ligand-receptor pairs between TIMP1+Epi cells and TIMP1-Epi cells as signaling cells and Treg cells. (PDF) [file pone.0335788.s003.pdf]
